# Multicenter Evaluation of a Gradient Diffusion Method for Antimicrobial Susceptibility Testing of Helicobacter pylori

**DOI:** 10.1128/spectrum.02111-21

**Published:** 2022-03-07

**Authors:** Salika M. Shakir, Joshua Otiso, George Keller, Hillary Van Heule, Lucas J. Osborn, Nicolynn Cole, Audrey N. Schuetz, Sandra S. Richter, Marc Roger Couturier

**Affiliations:** a Department of Pathology, University of Utah School of Medicine, Salt Lake City, Utah, USA; b Department of Laboratory Medicine, Cleveland Clinic, Cleveland, Ohio, USA; c Department of Laboratory Medicine and Pathology, Mayo Clinic, Rochester, Minnesota, USA; d ARUP Institute for Clinical and Experimental Pathology, Salt Lake City, Utah, USA; University of Arizona/Banner Health

**Keywords:** *Helicobacter pylori*, antimicrobial susceptibility, Etest, agar dilution, fastidious isolates organism, method comparison

## Abstract

Helicobacter pylori is an important human pathogen associated with peptic ulcer disease, dyspepsia, and gastric malignancy. Antimicrobial susceptibility testing (AST) is often requested for patients who fail eradication therapy. The Clinical and Laboratory Standards Institute (CLSI) reference method, agar dilution (AD), is not performed in most laboratories and maintaining organism viability during transit to a reference laboratory is difficult. We assessed the performance of the Etest (bioMérieux) as a method for H. pylori AST in comparison to AD. Etest MICs were determined for 83 H. pylori isolates at ARUP and Cleveland Clinic (CC). Categorical agreement (CA), very major, major, and minor errors (VME, ME, and mE) were determined for Etest using AD performed at Mayo Clinic Laboratories as the reference method. Testing on isolates with errors was repeated to determine final results summarized below. For clarithromycin, 66.3% of isolates were resistant (R) by AD; Etest results at each laboratory showed 1mE (1.2%) and 1 ME (3.8%). For tetracycline, only 2 isolates were R by AD; a single VME occurred at both sites (98.8% CA, 50% VME) with the same isolate. Applying EUCAST levofloxacin breakpoints to interpret ciprofloxacin results, 60.2% of isolates were R by AD; ARUP CA was 97.6% (1 ME (3%), 1 VME (2%)) and CC CA was 96.3% (1 ME (3%), 2 VMEs (4%)). Despite high error rates, the categorical agreement was acceptable (>90%) for all three antibiotics between AD and Etest. In-house susceptibility testing by gradient diffusion can allow for testing of fastidious organisms that may not survive transport to specialized laboratories; however, the method is not without technical challenges. Characterization of resistance mechanisms, increased AD dilutions, and testing from the same inoculum may determine if the observed errors reflect technical issues or breakpoints that need optimization.

**IMPORTANCE** Routine antimicrobial susceptibility testing (AST) of Helicobacter pylori by agar dilution is difficult to perform and not practical in most clinical microbiology laboratories. The Etest gradient diffusion method can be a reliable alternative for H. pylori AST with the advantage of being a less laborious quantitative method. This work reveals that an optimized Etest method can provide acceptable performance for H. pylori AST and describes the challenges associated with this methodology.

## INTRODUCTION

Helicobacter pylori causes gastritis and peptic ulcers as well as chronic, nonresolving infections associated with the development of gastric cancer. Current guidelines by the American College of Gastroenterology (ACG) for the management of H. pylori infection recommend that all patients receive eradication therapy to reduce the risk of ulcer bleeding. Triple therapy consisting of amoxicillin, a proton pump inhibitor (PPI), and clarithromycin or metronidazole for 14 days is recommended if the rate of antimicrobial resistance is below 15% (1). Quadruple therapy consists of a PPI, bismuth, tetracycline, and metronidazole for 14 days and is indicated for patients with previous macrolide exposure or severe penicillin allergies (1). An increase in H. pylori resistance to the standard antibiotics in the triple and quadruple therapies has led to frequent treatment failures (2–4) and as a result of these failures, levofloxacin-based-triple therapy (so called “salvage treatment”) has been advocated after initial success was reported in multiple studies (5–7). Fluoroquinolone resistance in H. pylori is also increasing which has decreased the efficacy of this salvage therapy (8). Clinicians caring for patients with treatment failure often submit gastric biopsy specimens to the microbiology laboratory with requests for H. pylori culture and susceptibility testing.

The Clinical and Laboratory Standards Institute (CLSI) has approved agar dilution as a “gold standard” reference AST method for H. pylori with established MIC breakpoints only for clarithromycin. Breakpoints for additional agents have been published by the European Committee on Antimicrobial Susceptibility Testing (EUCAST). Agar dilution testing is only available in a small number of laboratories, and the yield of send-out testing can be significantly hampered due to isolates losing viability in transit or becoming contaminated upon multiple subcultures and manipulations. The Etest (bioMérieux) is an MIC gradient diffusion method that has been described in various H. pylori studies to date but there is variability among testing procedures and reported assay performances. The purpose of this multicenter study was to assess the performance of Etest using a procedure optimized by members of the CLSI M45 subcommittee for H. pylori AST using agar dilution as the reference method (9, 10).

## RESULTS

An overview of the initial and final (postdiscrepancy analysis) categorical agreement and errors attributed to Etest for each antimicrobial agent by testing center is summarized in Table S1 (S1) and Table 1, respectively. Fig. S2 (A-C) shows the major errors and very major errors for each antimicrobial agent. Results for initial and discrepancy testing are outlined in Table S2.

**TABLE 1:** Final Etest performance for each sites measured as categorical agreement with agar dilution as reference method after discrepant analysis^*a*^

Antibiotic	Resistant by agar dilution	Categorical agreement	Minor errors	Major errors	Very major errors	Kappa coefficient	95% confidence interval	Site
Clarithromycin	66.3% (55/83)	97.6% (81/83)	1	1	0	0.96	0.9–1.0	CC, ARUP
Tetracycline	2.4% (2/83)	98.8% (82/83)	0	0	1	0.66	0.04–1.0	CC, ARUP
Ciprofloxacin	60.2% (50/83)	96.4% (80/83)	0	1	2	0.43	0.84–1.0	CC
		97.6% (81/83)	0	1	1	0.95	0.88–1.0	ARUP

a Kappa between 0.41 and 0.60: moderate agreement; Kappa between 0.61 and 0.80: substantial agreement; Kappa between 0.81 and 1.00: almost perfect agreement.

For clarithromycin (Tables 1 and 2), categorical agreement was 97.6% (81/83) after discrepancy testing with only 1 mE and 1 ME at each laboratory. Essential agreement (within ±1 doubling dilution) of final Etest MICs between laboratories occurred for 55.4% of isolates with clarithromycin (Table 2). Agar dilution results changed for 6 isolates with repeat clarithromycin testing and the final resistance rate was 66.3% (55/83) (Table 1 and Table S2).

**TABLE 2:** Clarithromycin MICs determined by Etest and the agar dilution method for 83 Helicobacter pylori isolates^*a*^

Etest MIC	No. isolates with reference agar dilution MIC (μg/mL)											
	**Initial ARUP**			**Repeat ARUP**			**Initial CC**			**Repeat CC**		
**(μg/mL)**	**≤0.25**	**0.5**	**>0.5**	**≤0.25**	**0.5**	**>0.5**	**≤0.25**	**0.5**	**>0.5**	**≤0.25**	**0.5**	**>0.5**
≤0.016	9						14		1 VME^*g*^			
0.03	7						6	1 mE^*b*^				
0.06	4						3					
0.12	4	1 mE^*b*^	1 VME^*d*^	1 CA^*e*^			1^*e*^	1 mE^*c*^	1 VME^*h*^			
0.25					1 mE^*c*^		1		1 VME^*f*^			
0.5					1 CA^*b*^					1 mE^*e*^	2 CA^*b*^^,^^*c*^	
1	1ME^*e*^	1 mE^*c*^							1			
2			3^*h*^						1			2 CA^*f*^^,^^*h*^
4						1 CA^*f*^			3			
8			5^*f*^						3			
16	1 ME^*i*^		5^*f*^						3			
32			10						2			
64			6						1			
128			1						2			1 CA^*g*^
256			1									
>256			23	1 ME^*i*^		2 CA^*d*^	1 ME^*i*^		36^*d*^	1 ME^*i*^		1 CA^*d*^
No. of isolates	26	2	55				26	2	55			

a Very major error (VME), major error (ME), minor error (mE), categorical agreement (CA), Cleveland Clinic (CC).

b Initial minor errors at both laboratories. Repeat Etest = AD result.

c Initial mE at both laboratories; repeat ARUP Etest of 0.25 μg/mL = mE. Repeat CC = AD result.

d Initial ARUP VME. Repeat ARUP Etest = AD result.

e Initial ARUP ME. Repeat ARUP was CA; repeat CC Etest was I (0.5 μg/mL) = mE.

f Repeat CC Etest 3 dilutions higher = CA.

g Initial CC VME. Repeat CC Etest yielded CA.

h Repeat CC Etest MIC result 4 dilutions higher & same MIC as ARUP initial result = CA.

i Initial ME at both laboratories repeated as R.

For tetracycline (Tables 1 and 3), only 2 isolates (2.4%) were resistant by agar dilution (MIC ≥2 μg/mL). One VME for the same isolate occurred at both laboratories and persisted with repeat testing for a final categorical agreement of 98.8% (82/83). An initial VME at ARUP resolved with repeat testing (Table S2). Essential agreement of final Etest MICs between laboratories was 87.9% for tetracycline (Table 3).

**TABLE 3:** Tetracycline MICs determined by Etest and the agar dilution method for 83 Helicobacter pylori isolates^*a*^

Etest MIC	No. isolates with reference agar dilution MIC (μg/mL)											
	**Initial ARUP**			**Repeat ARUP**			**Initial CC**			**Repeat CC**		
**(μg/mL)**	**≤0.06**	**1**	**≥2**	**≤0.06**	**1**	**≥2**	**≤0.06**	**1**	**≥2**	**≤0.06**	**1**	**≥2**
≤0.016	14	15					14	9				
0.03	7	14					11	16				
0.06	6	14					1	10				
0.12		9						9				
0.25		1					1	8	1 VME^*b*^			
0.5		1	1 VME^*b*^			1 VME^*b*^		2				1 VME^*b*^
1			1 VME^*c*^									
2						1 CA^*c*^			1			
No. of isolates	27	54	2				27	54	2			

a EUCAST breakpoints of susceptible (S) ≤1 μg/mL & resistant (R) >1 μg/mL were applied to determine very major error (VME), categorical agreement (CA).

b Initial and repeat VMEs at both laboratories.

c Initial VME at ARUP. Repeat Etest MIC increased 1 dilution = CA.

**FIG 1:**
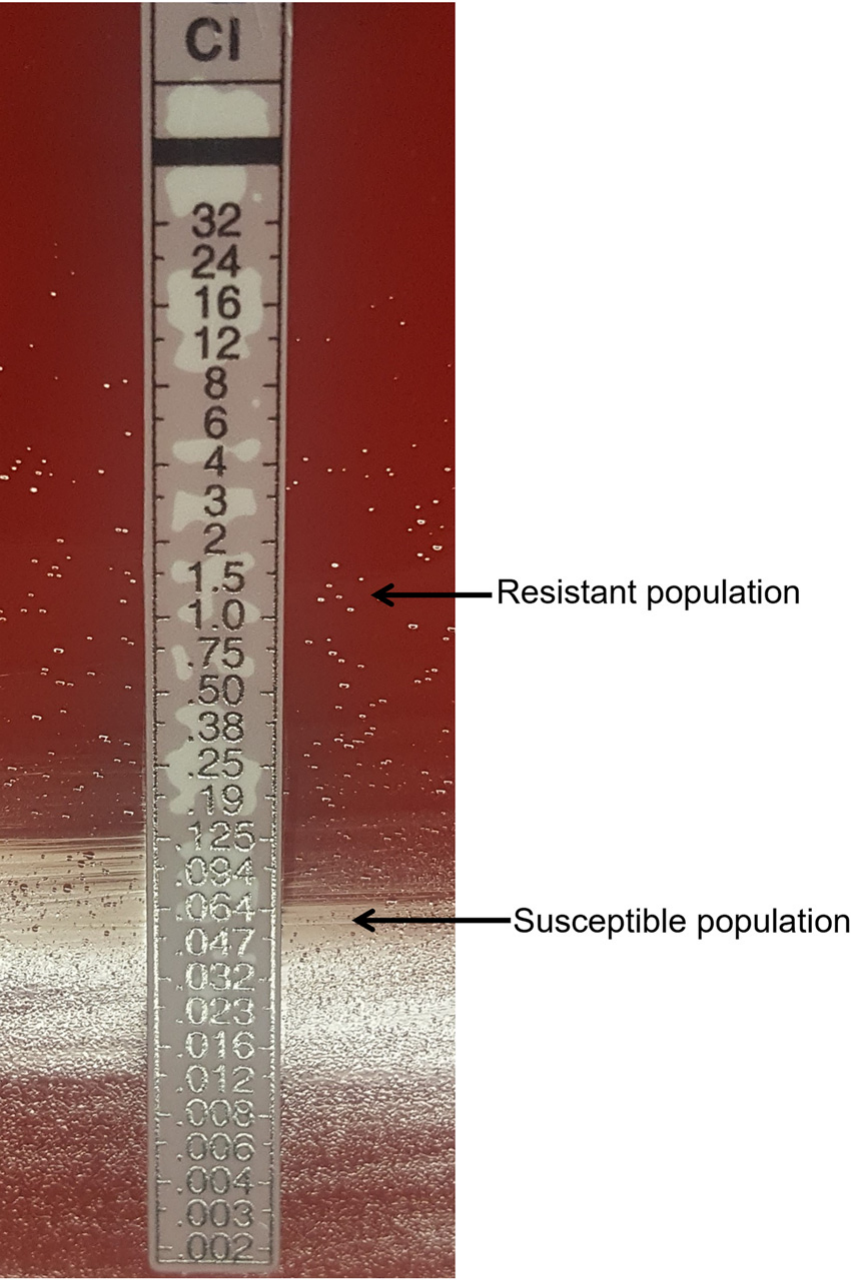
A H. pylori strain that appeared susceptible by agar dilution exhibited heteroresistance to ciprofloxacin by the Etest method at both ARUP and CC. The isolate was noted to have two subpopulations, one that was resistant (pinpoint colonies, MIC, >1 μg/mL) and another susceptible (MIC, 0.064 μg/mL) to ciprofloxacin when levofloxacin EUCAST breakpoints are applied.

For ciprofloxacin (Tables 1 and 4), agar dilution results interpretive categories changed for 3 isolates with repeat testing for a final resistance rate of 60.2%. The final Etest categorical agreement was 96.4% (80/83) at CC (1 ME, 2 VMEs) and 97.6% (81/83) at ARUP (1 ME, 1 VME). The ME was attributed to an isolate with two subpopulations, one that was susceptible (MIC 0.064 μg/mL) and one that was resistant (MIC >1 μg/mL) by the Etest method at both sites (Fig. 1). Three initial VMEs at CC and 1 VME at ARUP resolved with discrepancy testing (Table S2).

**TABLE 4:** Ciprofloxacin MICs determined by Etest and the agar dilution method for 83 Helicobacter pylori isolates^*a*^

Etest MIC	No. isolates with reference agar dilution MIC (μg/mL)							
	Initial ARUP		Repeat ARUP		Initial CC		Repeat CC	
**(μg/mL)**	**≤1**	**>2**	**≤1**	**>2**	**≤1**	**>2**	**≤1**	**>2**
0.008	2	1 VME^*b*^			3			
0.016	4				3	2 VME^*b*^^,^^*g*^		
0.03	7				9	1 VME^*f*^		1 VME^*b*^
0.06	12			1 VME^*b*^	10	2 VME^*h*^^,^^*i*^		1 VME^*h*^
0.12	6^*c*^				6			
0.25	2	1 VME^*d*^						
0.5		1 VME^*e*^			1			
1								
2		5^*g*^		1 CA^*g*^		2		
4		7				1		
8		3^*f*^		1 CA^*f*^		1		
16		2				1		1 CA^*f*^
32		2				3		
>32		28^*h*^^,^^*i*^	1 ME^*c*^	4 CA^*d*^^,^^*e*^^,^^*h*^^,^^*i*^	1 ME^*c*^	37^*d*^^,^^*e*^	1 ME^*c*^	4 CA^*d*^^,^^*e*^^,^^*g*^^,^^*i*^
No. of isolates	33	50			33	50		

a EUCAST levofloxacin breakpoints of susceptible (S) ≤1 μg/mL & resistant (R) >1 μg/mL were applied to determine very major (VM) & major errors (ME).

b Initial and repeat VMEs at both laboratories.

c Initial ME at CC; ME at both laboratories with repeat.

d Initial VME at ARUP, CA with repeat.

e Initial VME at ARUP; CA with repeat.

f Initial VME at CC; CA with repeat.

g Initial VME at CC; CA with repeat.

h Initial and repeat VME at CC.

i Initial VME at CC; CA with repeat.

## DISCUSSION

In this study, AST using Etest was compared at two study sites for a collection of previously phenotypically characterized isolates with harmonized testing protocols optimized by multiple members of the original CLSI M45-A3 working group. Etest, compared to agar dilution (AD), provides MIC values in-between 2-fold doubling dilutions, whereas agar dilution typically uses 2-fold doubling dilutions. Due to this disparity, we observed significant differences between results from the two methods for several isolates tested for the three drugs (Table S1). In addition, as AD employed limited dilutions tested at breakpoint concentrations, we were unable to calculate essential agreement between the two methods and clearly assess the accuracy of Etest. Overall, clarithromycin results correlated well between Etest and AD with results showing >90% categorical agreement before discrepancy resolution, and MIC’s being within ±1 log dilution after discrepancy resolution. Despite the low essential agreement for MIC values across the Etest testing sites and significant error rate, we observed substantial agreement (CA >95%) between Etest sites and AD for clarithromycin and tetracycline as observed by other groups strengthening the potential use of the Etest method for H. pylori AST (11, 12).

We observed that majority of the isolates tested in our patient population were resistant to clarithromycin (66.3%) and ciprofloxacin (60.2%). However, this does not reflect the national or regional resistance rates of H. pylori in the United States to either clarithromycin or fluoroquinolones which are estimated to be about 21%–30% (13). The higher resistance observed suggests a sampling bias toward patients undergoing esophagogastroduodenoscopy (EGD) and biopsy due to previously failed first and/or second line of therapy. Most (98.8%) of strains in our study tested susceptible to tetracycline by both AD and Etest. This is consistent with the low rates of tetracycline resistance (1–3%) that have been reported for H. pylori (14, 15). However, the low number of tetracycline resistant isolates in our study confound the comparison between AD and Etest methods.

One major limitation in our study was that both Etest and the comparative AD test were not performed in the same laboratory with the same inoculum. All isolates for Etest were stored at −80°C prior to testing, however, the age of the colonies and nonequivalent passage number of isolates selected may explain the discrepancy of the study results. The reproducibility between the initial and repeat AD results is also concerning and variability in inoculum used for the two methods across the three laboratories may be a factor. A second limitation of the study is the lack of levofloxacin AD results for comparison with Etest. While levofloxacin is used in fluoroquinolone-based therapies, susceptibility rates have been shown to be similar to ciprofloxacin (16). Further studies will be helpful to establish a direct comparison of Etest levofloxacin results with agar dilution.

Our study highlights the challenges and imprecisions when using AD as the gold standard method for H. pylori AST. The phenomenon of heteroresistance, described previously, (17, 18) may also contribute to the discrepancy of the results as independent subpopulations of H. pylori may have varied antibiotic susceptibility with resistant colonies potentially selected on multiple passages. This phenomenon may explain the three discrepancies observed in ciprofloxacin susceptibility testing since mixed populations of resistant and susceptible H. pylori colonies were observed for one isolate with the Etest method.

One of the main challenges for H. pylori AST testing is maintaining the viability of the organism during transport to a commercial laboratory; and preventing contamination of the cultures, both of which can delay antimicrobial susceptibility testing and reporting. Previous European studies demonstrate the ease of adopting Etest as an AST method with good correlation between Etest and agar dilution particularly for clarithromycin and amoxicillin (2, 11, 12, 18–21). However, the methodologies pertaining to different inoculum sizes, types of media used, and duration of incubation varied among different studies. Commercial laboratories lack a standardized testing protocol for H. pylori AST by Etest and rely on the manufacturer instructions for use that are not specific for H. pylori. Studies by Megraud et al. and Glupczynski et al. and our results from developmental studies (data not shown) demonstrate that the ideal testing conditions include inoculating a 3 McFarland of H. pylori uniformly on a sheep-blood supplemented MHA plate (aged ≥2 weeks), and incubation of the H. pylori AST cultures at 37°C for 72 h under microaerobic conditions (11, 19). These testing conditions yielded overall high categorical agreement with AD and high method reproducibility between Etest testing sites, supporting the adoption of this methodology by other clinical laboratories.

We did not test metronidazole as several studies have reported poor correlation between agar dilution and Etest and a lack of reproducible results for isolates tested in the same laboratory (18, 19, 22, 23). The Etest method overestimates the rate of metronidazole resistance by 10–20% compared to AD (18). Additionally, CLSI M45-A3 guidelines do not recommended *in vitro* testing for metronidazole as the resistance determination does not reliably predict treatment failure (9).

In conclusion, the performance of Etest using a standardized method shows substantial agreement with agar dilution for clarithromycin and tetracycline for H. pylori AST. A more direct comparison of Etest and agar dilution for levofloxacin is needed to further evaluate the utility of Etest to predict fluoroquinolone activity against H. pylori. Although challenging, in-house AST by gradient diffusion can allow results to be generated for fastidious organisms that may perish during transport and facilitate observation of heterogeneity that can be obscured when testing limited concentrations by agar dilution.

## MATERIALS AND METHODS

### H. pylori strains.

Frozen stocks of 83 H. pylori clinical isolates archived at ARUP Laboratories between 2013 and 2017 were used for the study under a protocol approved by University of Utah IRB. Stock cultures of isolates at ARUP with prior agar dilution results (*n* = 68) reported by Mayo Clinic Laboratories (MCL) were de-identified, prepared in 10% glycerol from the same plate, frozen at −70°C, and distributed to participating clinical microbiology laboratories (ARUP laboratories [ARUP] and Cleveland Clinic [CC]) for susceptibility testing by Etest. An additional 15 isolates without prior agar dilution results were distributed to MCL for susceptibility testing by AD and to ARUP and CC for susceptibility testing by Etest. Investigators performing the Etest method were blinded to the agar dilution results. Prior to testing, isolates were subcultured twice on Brucella agar supplemented with vitamin K and incubated for 72–96 h in a microaerobic atmosphere (10% CO_2_, 5% O_2_, and 85% N_2_) at 37°C until sufficient growth was observed.

### Etest method.

Mueller-Hinton agar with 5% sheep blood plates (150 mm diameter, BD) received at least 2 weeks earlier from the manufacturer were inoculated with a 3 McFarland suspension of the isolate prepared in brain heart infusion broth. Etest strips were applied, and the plates were incubated for 72 h in a GasPak (Becton, Dickinson) or Anoxomat (Advanced Instruments) system yielding a microaerobic environment (10% CO_2_, 5% O_2_, and 85% N_2_) at 37°C. A maximum of three Etest strips were placed on the surface of the plate to avoid overlaps between the elliptical zones of inhibition. The clinical isolates were tested with Etest strips of clarithromycin (0.016–256 μg/mL), tetracycline (0.016–256 μg/mL), and ciprofloxacin (0.006–32 μg/mL). The MIC was read as the intercept of the elliptical zone of inhibition (including pinpoint colonies within zone of inhibition) with the gradient strip and rounded up to the next 2-fold dilution (fig. S1). H. pylori strain ATCC 43504 was tested on each day of testing for quality control.

### Agar dilution method.

Testing of the H. pylori isolates was performed using the agar dilution method according to CLSI guidelines (9). Briefly, a saline suspension of a 2 McFarland standard was prepared from a 72-h culture of the isolate. A 3 μl inoculum was spotted on Mueller-Hinton agar with 5% aged sheep blood (≥2-weeks) containing the respective antibiotic (clarithromycin 0.25 μg/mL and 0.5 μg/mL; ciprofloxacin 1 μg/mL and 2 μg/mL; tetracycline 0.06 μg/mL, 1 μg/mL, and 2 μg/mL) using a replicator device. The plates were incubated at 37°C for 72 h in a microaerobic atmosphere. The MICs were read as the lowest antibiotic concentration that completely inhibited visible growth.

### Data and statistical analysis.

Results were interpreted by applying CLSI breakpoints for clarithromycin: susceptible (S), ≤0.25 μg/mL; intermediate (I), =0.5 μg/mL; resistant (R), >0.5 μg/mL). The EUCAST breakpoints for tetracycline (S, ≤1 μg/mL; *R* >1 μg/mL) were applied. EUCAST breakpoints for levofloxacin (S, ≤1 μg/mL; R, >1 μg/mL) were applied to ciprofloxacin results, due to lack of CLSI or EUCAST breakpoints for ciprofloxacin and the limited AD concentrations tested. Categorical agreement (CA), very major errors (VME), major errors (ME), minor errors (mE), and Cohen’s kappa were determined for Etest using agar dilution as the reference method. The denominator for VME rate calculation was the number of resistant isolates; the denominator for ME rate was the number of susceptible isolates. Discrepancy analysis was performed by repeating agar dilution and Etest on isolates with VME, ME, and mE errors compared to initial agar dilution results. Frozen stocks of 18 isolates with discordant AD and Etest results were prepared from individual stock cultures, frozen in 10% glycerol, and distributed to the participating laboratories. Isolates at ARUP and CC were tested in duplicate by Etest from the same inoculum. The repeat agar dilution results served as the comparator for the repeat Etest results. Etest MIC results were same between ARUP and CC and within the two sites for 17 out of 18 isolates.
