# Supplementary material for: Multicenter Evaluation of a Gradient Diffusion Method for Antimicrobial Susceptibility Testing of Helicobacter pylori
Source: Microbiol Spectr. 2022 Mar 7;10(2):e02111-21. doi: 10.1128/spectrum.02111-21 (PMC9045198; doi:10.1128/spectrum.02111-21)

Supplementary Data

Supplementary Table 1. Initial Etest performance for each sites measured as categorical agreement with agar dilution as reference method

Antibiotic	Resistant by agar dilution	Categorical agreement	Minor Errors	Major Errors	Very Major Errors	Site
Clarithromycin	62.6% (52/83)	92.8% (77/83)	2	2	3	CC
		94% (78/83)	2	1	1	ARUP
Tetracycline	2.4% (2/83)	98.8% (82/83)	0	0	1	CC
		97.6% (81/83)	0	0	2	ARUP
Ciprofloxacin	57.8% (48/83)	92.8% (77/83)	0	1	5	CC
		96.4% (80/83)	0	0	3	ARUP

Supplementary Table 2. Breakdown of discrepant *H. pylori* isolates with initial and repeat MIC values for clarithromycin, tetracycline, and ciprofloxacin.

		MIC µg/mL				MIC µg/mL	
**Antibiotic**	**Isolate #**	**Agar Dilution**	**ARUP initial result**	**CC initial result**		**ARUP repeat result**	**CC repeat result**
**Clarithromycin**	2	0.5 (I)	1 (mE)	0.125 (mE)		0.25 (mE)	0.5
	9	≤0.25 (S)	1 (ME)	0.125		0.125	0.5 (mE)
	21	> 0.5 [R]	2	0.125 (VME)		2	2
	34	> 0.5 [R]	8	0.25 (VME)		4	4
	67	> 0.5 [R]	16	≤0.016 (VME)		not tested	128
	69	0.5 (I)	0.12 (mE)	0.032 (mE)		0.5	0.5
	84	>0.5 [R]	0.125 (VME)	>256		>256	>256
	86	≤0.25 (S)	16 (ME)	>256 (ME)		>256 (ME)	>256 (ME)
**Tetracycline**	69	>2	1 (VME)	2		2	not tested
	87	2	0.5 (VME)	0.25 (VME)		0.5 (VME)	0.5 (VME)
**Ciprofloxacin**	13	>2	>32	0.064 (VME)		>32	0.064 (VME)
	29*	≤1	0.12	>32 (ME)		>32 (ME)	>32 (ME)
	31	>2	0.5 (VME)	>32		>32	>32
	49	>2	2	0.016 (VME)		not tested	>32
	71	>2	8	0.032 (VME)		>32	16
	76	>2	>32	0.064 (VME)		not tested	>32
	77	>2	0.008 (VME)	0.016 (VME)		0.032 (VME)	0.032 (VME)
	112	>2	0.25 (VME)	>32		>32	>32

*This isolate was noted to have two subpopulations by Etest, one that was resistant (MIC, 32 µg/mL) and another susceptible (MIC, 0.064 µg/mL) to ciprofloxacin when levofloxacin EUCAST breakpoints are applied.

Supplemental Figure 1. Image of a Clarithromycin Etest MIC read at >0.5 µg/mL when clarithromycin CLSI breakpoints are applied susceptible ≤0.25 µg/mL; intermediate 0.5 µg/mL; resistant >0.5 µg/mL.

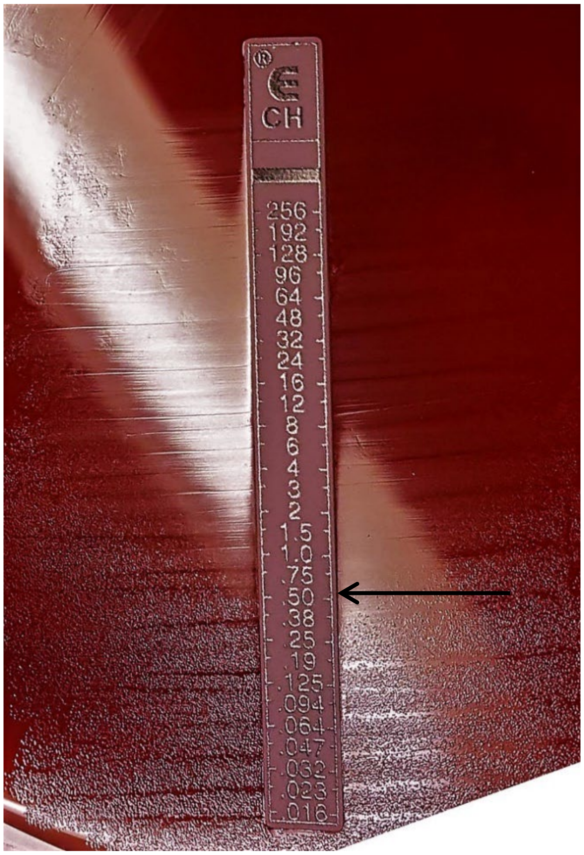

Supplemental Figure 2A: Percent of Clarithromycin resistant isolates with minor error, major error, and very major error in comparison with agar dilution.

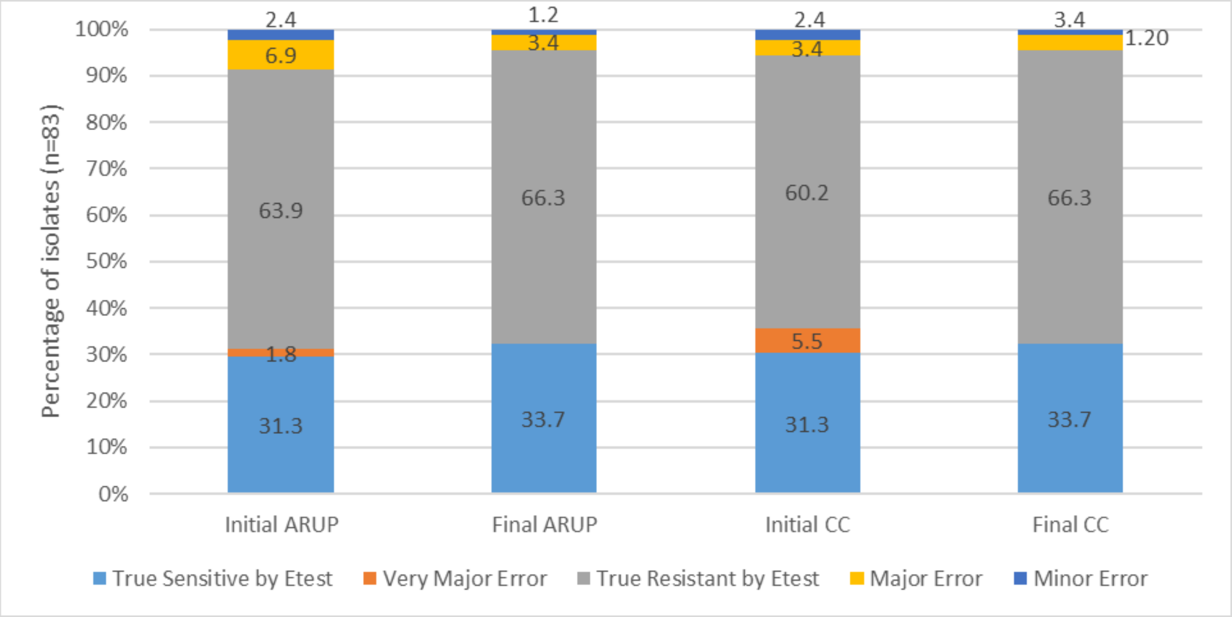

Supplemental Figure 2B: Percent of Tetracycline resistant isolates with very major error in comparison with agar dilution.

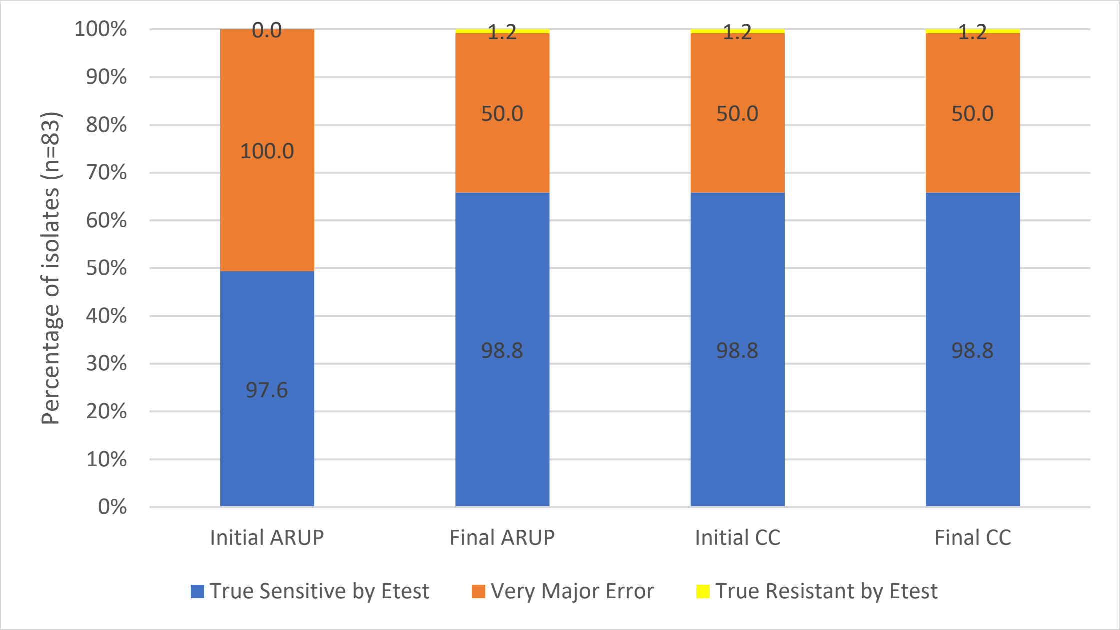

Supplemental Figure 2C: Percent of Ciprofloxacin resistant isolates with major and very major error in comparison with agar dilution.

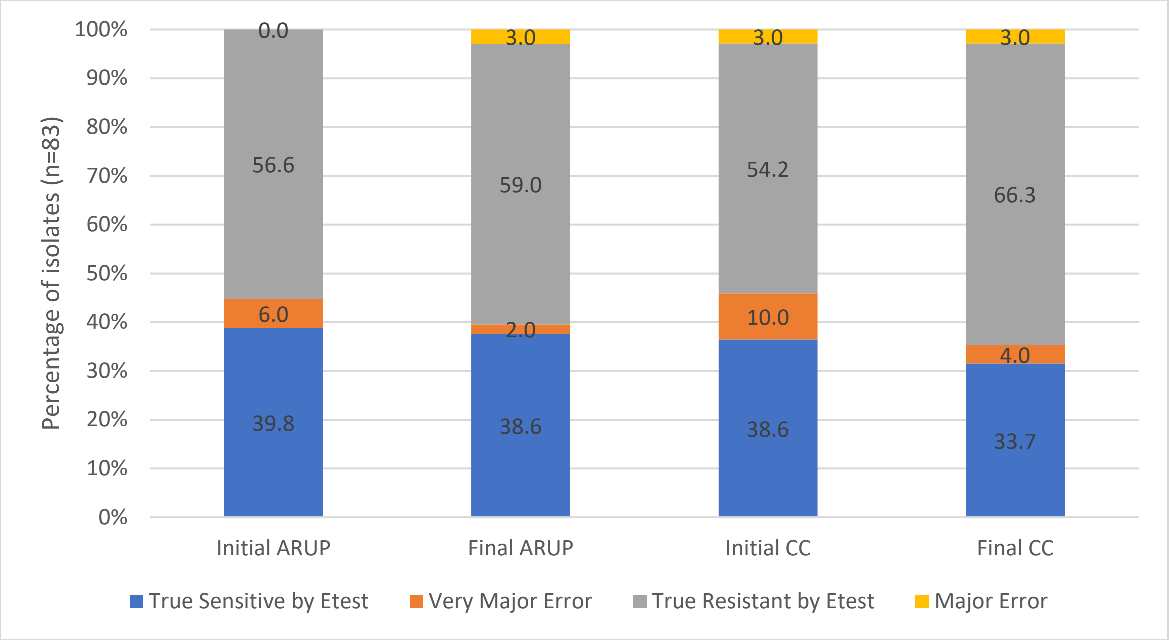

Supplement: SUPPLEMENTAL FILE 1 — Supplemental material. Download SPECTRUM02111-21_Supp_1_seq5.pdf, PDF file, 0.3 MB [file spectrum02111-21_supp_1_seq5.pdf]
